# Investigating time to first birth among women of reproductive age in Bangladesh: a survival analysis of nationwide cross-sectional survey data

**DOI:** 10.1186/s41043-023-00492-1

**Published:** 2024-01-02

**Authors:** Abdus Sobhan, Mohammed Moinuddin, Md. Moyazzem Hossain

**Affiliations:** 1 Chief Economist’s Unit, Bangladesh Bank, Head Office, Dhaka, 1000 Bangladesh; 2 School of Medicine and Dentistry, University of Central Lancashire, Preston, Lancashire, PR1 2HE UK; 3 Department of Statistics and Data Science, Jahangirnagar University, Savar, Dhaka, 1342 Bangladesh

**Keywords:** Age at first birth, Bangladesh, Factors, Reproductive age women

## Abstract

**Background:**

The birth of the first child is an important turning point in a woman’s life as it is the starting point of the demanding responsibilities of motherhood and childcare. This study aimed to explore the waiting time and the significant indicators of time to the first birth of aged 15–49 years of ever-married women in Bangladesh.

**Methods:**

The study considered the most recent country-representative data collected from Bangladesh Demographic and Health Survey (BDHS) in 2017/18. The log-rank test was used to assess the statistical significance of the observed difference between waiting time to first birth and various socio-economic and demographic factors. The Cox proportional hazard model is applied to identify the influential factors for waiting time to first birth.

**Results:**

About 55% of the respondents’ age at their first birth was less than 18 years. More than 21% of them were 20 years and above at their first birth. Findings revealed a higher mean age at first birth in urban areas than in rural areas. Also, in Dhaka and Sylhet region, women have a higher age at first than in other regions of Bangladesh. Results show that the place of residence, region, age at first marriage, age at first sex, respondent’s education, employment status, contraceptive use, and mass media exposure were found to be statistically significant determinants of the age of respondents at the time of first birth. Findings also show that a woman from rural areas was likely to be 5% smaller in age at the time of first birth than their counterpart (aHR 1.05; 95% CI 1.01–1.10). The age at first birth of a woman in Chattogram was 24% shorter, while in Rangpur and Barishal, that age was increased by 14% and 8%, respectively. A woman with no education, primary, and secondary education had 28%, 38%, and 29%, respectively, shorter age at first birth than that of the higher educated women. Mass media unexposed women were shorter aged at first birth by 27% (aHR 1.27; 95% CI 1.10–1.47) compared to the women who were mass media exposed.

**Conclusion:**

It is necessary to increase the age of mothers at first birth which may help to reduce the prevalence of child marriage in Bangladesh. The study findings will be helpful to the policymakers in identifying the gap and designing the programmes targeting the early timing of first birth to reduce child mortality as well as poor maternal outcomes which will be beneficial for achieving the Sustainable Development Goal-3 in Bangladesh.

## Background

The birth of the first child is an important turning point in a woman’s life as it is the starting point of the demanding responsibilities of motherhood and childcare [1, 2]. It has a direct association with fertility and plays a crucial role in the future life of each individual woman [3]. The first birth’s timing affects how many children a woman has, and women who give birth earlier in life have more children than those who do so later [4, 5] which, in turn, leads to a growth of population [6, 7]. On the other hand, delaying pregnancy and childbirth among young females may help them achieve higher levels of education, which could lead to positive social consequences, such as better health and greater well-being in general for their children [8]. In developing countries, the social and economic consequences of early age pregnancy and early childbearing are more frequent, which is allied with low birth weight, maternal mortality, truncated education, and productivity, and consequently intergenerational poverty transmission [4, 9, 10]. Early age at first birth is linked to chronic illnesses such as diabetes, osteoporosis, arthritis, chronic obstructive pulmonary disease, coronary heart disease, high blood pressure, stroke, and cancer [11–13]. At the same time, miscarriage, chromosomal defects, numerous pregnancies, diabetes mellitus, hypertension, low birth weight, preterm birth, breast cancer, and maternal mortality are all connected to advanced age (> 30 years) at first birth [14–16].

Previous research has found that a woman's age, education, place of residence, employment status, and contraceptive use are all linked to the age at which she gives her first birth in Indonesia [17]. A previous study found that regional variations exist, particularly in Sylhet and Chittagong, where total fertility rates are significantly higher than the national average. Women in Sylhet are far more likely than women in other divisions in Bangladesh to have a third or fourth child sooner [18]. Another study found that women in the Chattogram and Sylhet divisions had significantly shorter birth intervals than women in other parts of Bangladesh [19] and that may be related to less tendency of using contraceptive methods [20]. Women's marriage decisions are heavily influenced by factors such as education and where they reside [21]. A previous study highlighted that educational level is significantly associated with the age at first birth [17] and education has an impact on women’s marriage decisions [22]. Lower-income women are more likely to start childbearing earlier, have more children, and experience more difficulties during childbirth [23]. Meanwhile, women who started getting pregnant too early without any career development may discover themselves unemployed in the future [24]. Women who became pregnant in their adolescent period (aged 15–19 years) may lead to the termination of schooling, unemployment, poor maternal and child health outcomes, an increase in the number of children per woman, gender inequity, and destitution of young mothers and their families, as well as the community at large [25–30]. Moreover, women aged less than 18 years are sometimes pressured by peer groups, which may raise their chances of becoming pregnant early [21, 31]. In addition, family planning may postpone childbearing, and women who use contraceptives are more likely to have a late first childbirth than women who do not [21]. The inability to find a suitable partner to marry or the inability to pay for expensive wedding ceremonies or dowries may extend the age at first marriage that prolonged the age at first child [32, 33].

In modern times, many children are born before their mothers’ marriage, posing significant health hazards such as abortion and HIV [34]. Moreover, one of the important goals of the United Nations adopted global Sustainable Development Goals (SDGs) is to ensure healthy lives, promote well-being for people of all ages, and reduce child and maternal mortality. Bangladesh’s government is also determined to achieve these goals by 2030 [35]. Some research highlighted that becoming a first-time mother before the age of 20 years is likely to increase mortality [36–38]. Therefore, it is important to increase the mean age of women at first birth which may reduce the mortality rates and help achieve the SDGs. Early childbearing increases overall fertility and population growth. It has been associated with both maternal and child morbidity and mortality. However, limited literature is available focusing on this issue considering the most recent BDHS-2017/18 survey in Bangladesh.

The BDHS-2017/18 report suggests that there have been many changes in various health indicators during the time from 2014 to 2017 [39]. Therefore, the current analysis of the latest nationwide dataset will add valuable information about the influential factors of the age at first birth in Bangladesh. The authors expect that the findings of this investigation will update the understanding of the current age at first birth and its influential factors in Bangladesh. This eventually could help policymakers to take plan for future interventions to lessen the total fertility rate and reduce the consequences of first childbearing at an early age. Therefore, the focus of this research is to determine the influential factors of time to first birth among ever-married women in Bangladesh.

## Materials and methods

### Study design and settings

To find the per cent distribution of women’s different socio-economic and demographic characteristics and for subsequent analysis, this study considers the most recent country-representative data collected from BDHS-2017/18. The sampling frame of this survey was the list of enumeration areas (EAs) of the 2011 Population and Housing Census of the People's Republic of Bangladesh. The primary sampling unit of this survey was an EA. The survey used a two-stage stratified cluster sampling technique. In the first stage, 675 EAs were chosen, with 227 and 448 EAs from urban and rural areas. However, data were not possible to collect from 3 EAs due to a natural disaster. These clusters were in Dhaka (one urban cluster), Rajshahi (one rural cluster), and Rangpur (one rural cluster). In the second stage of sampling, a systematic sample of 30 households was selected from each selected EA. A total of 20,250 residential households were selected for this survey. Among the 20,376 ever-married women aged 15–49 years eligible for interviews, 20,127 were interviewed, yielding a response rate of around 99%. After discarding the missing data, a total of 18,134 women were included in the final analysis. A weighted sample is used for this analysis. The sampling procedure in detail is available in the report of the Bangladesh Demographic and Health Survey-2017/18 [39].

### Outcome variable

The outcome variable in this study was the age in years at first birth. Once a woman gives their first birth it is considered as an event.

### Covariates

The potential determinants used in this study were motivated by the availability in the BDHS dataset, field expertise and relevant literature [2, 19, 21, 30, 40–44]. The socio-demographic factors, husband-related factors, reproductive factors, media exposure, etc., are included as potential determinants of age at first birth in the analysis. A complete list with summaries of these variables is presented in Table 1.

**Table 1:** Percentage distribution of the participants and average age at first birth by socioeconomic and demographic characteristics

Background characteristics	Overall		Age at first birth (years)	*p* value of Log-rank test
	No. of women	Percentage	Mean (SE)	
Overall	18,134	100.0	18.2 (0.024)	
Place of residence				
Urban	6595	36.37	18.70 (0.045)	< 0.001
Rural	11,539	63.63	17.86 (0.028)	
Religion				
Muslim	16,299	89.88	18.05 (0.025)	< 0.001
Non-Muslim	1835	10.12	19.17 (0.081)	
Administrative region				
Barishal	1952	10.76	18.11 (0.074)	< 0.001
Chattogram	2622	14.46	18.11 (0.057)	
Dhaka	2623	14.46	18.54 (0.069)	
Khulna	2358	13	18.00 (0.068)	
Mymensingh	1948	10.74	18.03 (0.075)	
Rajshahi	2327	12.83	17.71 (0.066)	
Rangpur	2288	12.62	17.66 (0.068)	
Sylhet	2016	11.12	19.24 (0.076)	
Mother’s current age (years)				
Mean (SD)	32.66 (8.78)			
< 30	7266	40.07	17.86 (0.031)	< 0.001
30–39	6276	34.61	18.31 (0.045)	
40 or more	4592	25.32	18.45 (0.056)	
Age at marriage (Years)				
< 15	6025	33.38	15.85 (0.030)	< 0.001
15–17	7658	42.43	17.97 (0.024)	
18 or more	4365	24.19	21.73 (0.046)	
Age at first sex (years)				
< 15	5620	31	15.81 (0.032)	< 0.001
15–17	7723	42.6	17.82 (0.024)	
18 or above	4787	26.4	21.47 (0.045)	
Respondent’s education				
No education	3084	17.01	17.49 (0.057)	< 0.001
Primary	6001	33.09	17.44 (0.037)	
Secondary	6865	37.86	18.02 (0.034)	
Higher	2184	12.04	21.56 (0.082)	
Husband’s current age (years)				
Mean (SD)	40.85 (10.73)			
< 40	7986	46.87	18.12 (0.033)	< 0.001
40–49	4855	28.49	18.39 (0.052)	
50 or more	4198	24.64	18.10 (0.054)	
Husband’s education				
No education	3828	22.45	17.43 (0.048)	< 0.001
Primary	5450	31.96	17.54 (0.038)	
Secondary	4918	28.84	18.14 (0.042)	
Higher	2818	16.53	20.54 (0.075)	
Respondent’s employment status				
Non-employment	9082	50.08	18.44 (0.035)	< 0.001
Employment	9052	49.92	17.89 (0.034)	
Wealth index				
Poorest	3537	19.5	17.43 (0.048)	< 0.001
Poorer	3494	19.27	17.62 (0.047)	
Middle	3528	19.46	17.77 (0.051)	
Richer	3635	20.05	18.25 (0.054)	
Richest	3940	21.73	19.58 (0.061)	
Use of contraception				
Non-user	6769	37.33	18.30 (0.042)	< 0.001
User	11,365	62.67	18.08 (0.03)	
Mass media exposure				
No	18,032	99.44	18.14 (0.024)	< 0.001
Yes	101	0.56	21.90 (0.455)	
Husband’s occupation				
Did not work	1,459	8.05	17.89 (0.088)	< 0.001
Professional/technical/managerial	1,436	7.93	20.85 (0.114)	
Sales	3,242	17.89	18.24 (0.057)	
Agricultural—self-employed	1,563	8.63	17.55 (0.074)	
Agricultural—employee	2,762	15.24	17.59 (0.056)	
Services	1,903	10.5	18.02 (0.068)	
Skilled manual	5,683	31.37	18.01 (0.040)	
Others	70	0.39	18.07 (0.456)	
Husband’s desire for children				
Both want same	12,486	78.24	18.31 (0.030)	< 0.001
Husband wants more	1910	11.97	17.86 (0.074)	
Husband wants fewer	1019	6.39	18.12 (0.102)	
Don't know	544	3.41	17.82 (0.141)	

### Statistical analysis

The summary statistics count with percentage for categorical and mean for continuous variables are used to describe the dataset. The distribution of age at first birth is presented in a histogram. Kruskal–Wallis H test is used to assess the statistical significance of mean age at first birth among different covariate groups [45]. Survival analysis is conducted to serve the main objectives of this study considering ‘first birth’ as the event and age at ‘first birth’ as the waiting time. Kaplan–Meier’s survival probabilities are estimated and presented using survival curves [46]. Log-rank test is employed to test the statistical difference in survival curves among different covariate groups [47].

To determine the factors influencing the age at first birth, the Cox proportional hazard (Cox PH) model is employed [48]. The proportional hazard (PH) assumption was verified using both a graphic and a global test. All the candidate determinants that showed a statistical significance with a 20% level of significance either in log-rank or Kruskal–Wallis H test are included in the Cox PH model. The authors choose this cut-off value for possible inclusion of all relevant variables in the model. The authors motivated this by previous studies [49–51]. The final list of variables is selected using stepwise procedure, and the results of the final model are reported in the results section. All the analyses are carried out using the statistical software package STATA, version 14.0.

## Results

**Fig. 1:**
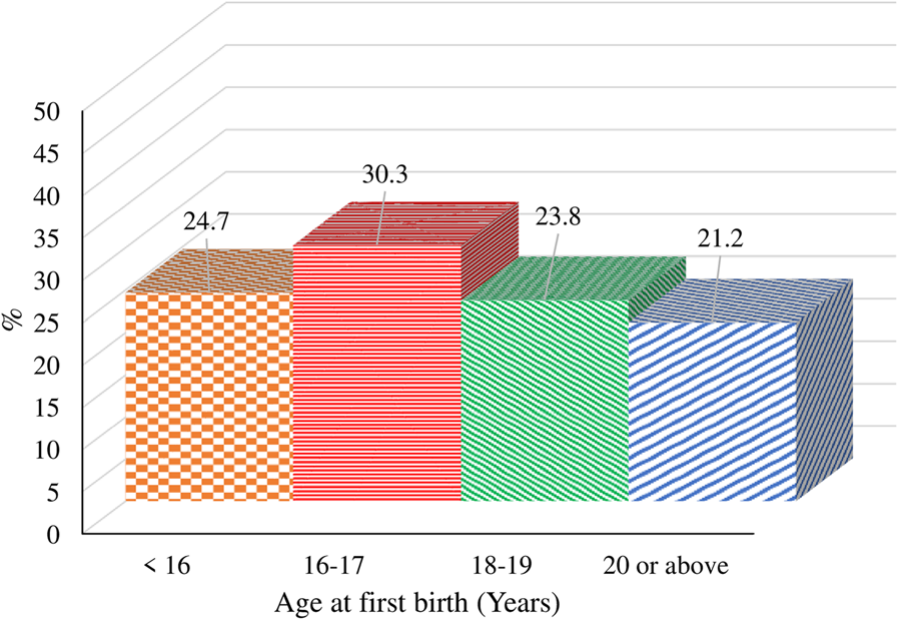
Respondent’s age at first birth (in years)

A total of 18,134 married women, who had first birth, were interviewed in 2017–18. More than half (55%, *n* = 26,280) of the respondents’ age at their first birth was less than 18 years and about 21% (*n* = 10,144) of them were 20 years and above at their first birth (Fig. 1).

About two-thirds (63.6%) of the respondents were from rural areas, 17.0% had no education, 50.1% were not employed, 62.7% were contraceptive users, and 40.1%, 34.6%, and 25.3% were aged less than 30 years, 30–39 years, and more than 40 years, respectively. About three-fourth (75.8%) of the respondents were aged less than 18 years at their marriage, of which 33.4% were even less than 15 years. The mean age at first birth was 18.2 years. Analysis revealed the higher mean age at first birth in the urban area (18.7 years), in Dhaka (18.54 years) and Sylhet region (19.24 years), who belong to higher educated group (20.54 years), who are from the richest families (19.58 years) and who had media exposure (21.9 years) in compared to the others group of the respondents (Table 1).

The authors checked the multicollinearity among covariates using the variance inflation factor (VIF) for each independent variable. Most of the variables have a value of VIF less than 5. However, the values of VIF for age at marriage and age at first sex are 5.021 and 6.048, respectively, which suggest moderate multicollinearity. After adjusting all selected variables in the Cox-proportional hazard model, it is found that the place of residence (*p* < 0.05), region, age at first marriage (*p* < 0.001), age at first sex (*p* < 0.001), respondent’s education (*p* < 0.001), employment status (*p* < 0.05), contraceptive use (*p* < 0.001), and mass media exposure (*p* < 0.001) were found as statistically significant determinants of the age of respondents at the time of first birth (Table 2). Analysis of the adjusted hazard ratio (aHR) revealed that a woman from a rural area was likely to be 5% smaller in age at the time of first birth than their counterpart (aHR 1.05; 95% CI 1.01–1.10). The age at first birth of a woman in Chattogram was 24% shorter, while in Rangpur and Barishal, that age was increased by 14% and 8% respectively. Respondents’ age less than 15 years at marriage also decreased the shorter time to first birth 1.7 times than the women who were aged more than 18 years at marriage. According to the educational level of the respondents, a woman with no education, primary, and secondary education had 28%, 38%, and 29%, respectively, shorter age at first birth than that of the higher educated women. A woman whose husband with primary education had the shortest (18%) age at first birth compared to the women whose husband was higher educated. Employed women had only five per cent (aHR 1.05; 95% CI 1.01–1.10) shorter age at first birth than the non-employed women. Contraceptive user and mass media unexposed women were shorter aged at first birth by 18% (aHR 1.18; 95% CI 1.13–1.23) and 27% (aHR 1.27; 95% CI 1.10–1.47), respectively, compared to the women who were contraceptive non-user and mass media exposed (Table 2).

**Table 2:** Proportional hazard model on the determinants of time to first birth for the significant socioeconomic and demographic covariates, BDHS 2017–18

Background characteristics	Number of women *N* = 18,134			Number of women *N* = 15,826		
	Unadjusted HR	*p* value	95% CI	Adjusted HR	*p* value	95% CI
*Place of residence*						
Rural	1.25	< 0.001	(1.21,1.28)	1.05	0.025	(1.01,1.10)
Urban	Ref.			Ref.		
*Religion*						
Muslim	1.29	< 0.001	(1.24,1.34)	1.00	0.875	(0.95,1.05)
Non-Muslim	Ref.			Ref.		
*Region*						
Barishal	1.32	< 0.001	(1.25,1.39)	1.08	0.056	(1.00,1.17)
Chattogram	1.30	< 0.001	(1.24,1.36)	1.24	< 0.001	(1.16,1.32)
Dhaka	1.16	< 0.001	(1.10,1.21)	1.04	0.283	(0.97,1.11)
Khulna	1.35	< 0.001	(1.29,1.42)	1.01	0.708	(0.94,1.10)
Mymensingh	1.33	< 0.001	(1.26,1.40)	0.94	0.176	(0.86,1.03)
Rajshahi	1.44	< 0.001	(1.37,1.52)	1.06	0.143	(0.98,1.14)
Rangpur	1.50	< 0.001	(1.43,1.59)	1.14	0.001	(1.06,1.24)
Sylhet	Ref.			Ref.		
*Mother’s current age (years)*						
Less than 30	1.20	< 0.001	(1.16,1.24)	1.72	< 0.001	(1.57,1.88)
30–39	1.05	0.008	(1.01,1.10)	1.29	< 0.001	(1.19,1.40)
40 +	Ref.			Ref.		
*Age at marriage (Years)*						
< 15	5.20	< 0.001	(4.91,5.50)	5.08	< 0.001	(4.78,5.40)
15–17	2.51	< 0.001	(2.42,2.59)	2.37	< 0.001	(2.29,2.45)
18+	Ref.			Ref.		
*Age at first sex (Years)*						
< 15	4.88	< 0.001	(4.60,5.17)	4.87	< 0.001	(4.57,5.19)
15–17	2.46	< 0.001	(2.38,2.54)	2.35	< 0.001	(2.27,2.43)
18+	Ref.			Ref.		
*Respondent’s education*						
No education	2.27	< 0.001	(2.15,2.39)	1.28	< 0.001	(1.17,1.40)
Primary	2.40	< 0.001	(2.30,2.50)	1.38	< 0.001	(1.30,1.47)
Secondary	2.08	< 0.001	(2.00,2.16)	1.29	< 0.001	(1.23,1.35)
Higher secondary or above	Ref.			Ref		
*Husband’s current age (Years)*						
Less than 40	1.02	0.246	(0.98,1.06)	0.93	0.127	(0.85,1.02)
40–49	0.96	0.046	(0.92,1.00)	1.01	0.812	(0.93,1.09)
50 +	Ref.			Ref.		
*Husband’s education*						
No education	1.98	< 0.001	(1.89,2.08)	1.15	0.001	(1.06,1.25)
Primary	1.95	< 0.001	(1.86,2.03)	1.18	< 0.001	(1.10,1.26)
Secondary	1.68	< 0.001	(1.61,1.75)	1.14	< 0.001	(1.08,1.21)
Higher	Ref			Ref.		
*Respondent’s employment status*						
Yes	1.13	< 0.001	(1.10,1.16)	1.05	0.016	(1.01,1.10)
No	Ref.			Ref.		
*Wealth index*						
Poorest	1.61	< 0.001	(1.54,1.69)	0.96	0.301	(0.89,1.04)
Poorer	1.56	< 0.001	(1.49,1.62)	0.96	0.284	(0.90,1.03)
Middle	1.47	< 0.001	(1.4,1.53)	0.97	0.385	(0.91,1.04)
Richer	1.31	< 0.001	(1.25,1.37)	0.96	0.129	(0.90,1.01)
Richest	Ref.			Ref.		
*Use of contraception*						
User	1.07	< 0.001	(1.04,1.10)	1.18	< 0.001	(1.13,1.23)
Non-user	Ref.			Ref.		
*Mass media exposure*						
No	1.95	< 0.001	(1.68,2.27)	1.27	0.001	(1.10,1.47)
Yes	Ref.			Ref.		
*Husband’s occupation*						
Professional/technical/managerial	0.56	< 0.001	(0.52,0.60)	0.91	0.226	(0.78,1.06)
Sales	0.93	0.03	(0.87,0.99)	0.95	0.539	(0.82,1.11)
Agricultural—self-employed	1.12	0.002	(1.04,1.21)	0.93	0.388	(0.79,1.10)
Agricultural—employee	1.10	0.007	(1.03,1.17)	0.98	0.796	(0.84,1.14)
Services	0.97	0.395	(0.91,1.04)	0.94	0.409	(0.80,1.09)
Skilled manual	0.99	0.747	(0.93,1.05)	0.96	0.563	(0.82,1.11)
Others	0.94	0.578	(0.76,1.16)	0.89	0.434	(0.67,1.19)
Did not work	Ref.			Ref.		
*Husband’s desire for children*						
Husband wants more	1.12	< 0.001	(1.07,1.17)	1.08	0.016	(1.01,1.15)
Husband wants fewer	1.05	0.106	(0.99,1.12)	1.02	0.553	(0.95,1.10)
Both want same	Ref.			Ref.		

Ref.: Reference category

**Fig. 2:**
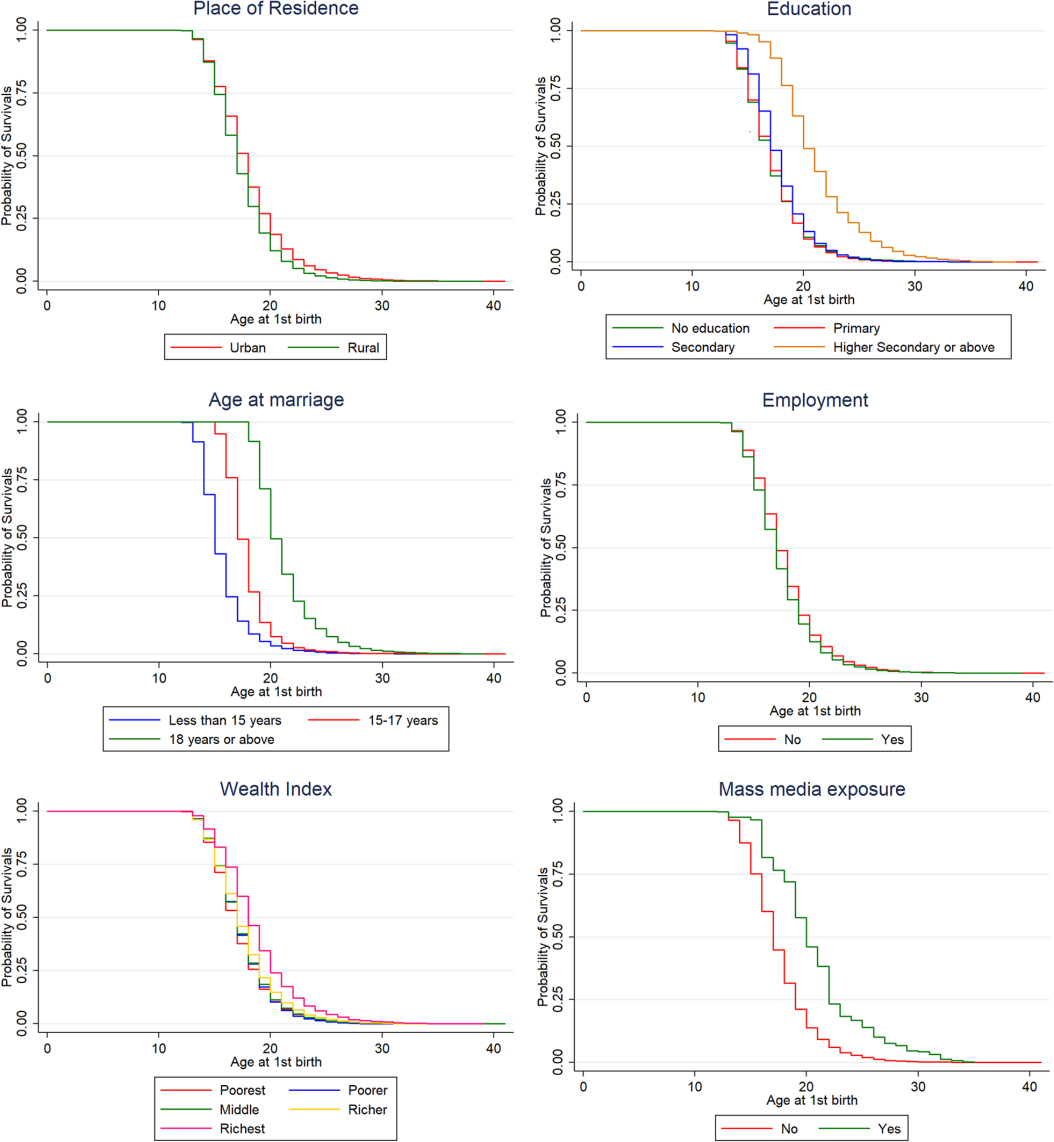
Kaplan–Meier survival curves of timing at first birth by the selected respondents characteristics

Figure 2 illustrates the survival curves of age at first childbirth by the selected respondents’ characteristics. It shows the probability that the first childbirth has occurred at age *t*. The survivorship function pattern generally showed that the group defined by the upper curve had a longer survival than the group defined by the lower curve. For instance, women who live in rural areas are more likely than those who reside in urban areas to delay having their first child. Women with higher educational achievement continuously have lower odds of having their first child at a younger age than women with lower educational attainment (Fig. 2).

## Discussion

This study used survival analysis techniques to explore the factors influencing the timing of first birth among reproductive age Bangladeshi women after marriage based on the BDHS 2017–18 data. It is important to note that the age at first birth can have significant implications for both maternal and child health. Women who give birth at a younger age may be at increased risk of complications during pregnancy and childbirth, and their children may also be at higher risk of poor health outcomes. In Bangladesh, the average age at first birth among women of reproductive age may vary depending on various factors such as education, socio-economic status, and access to family planning services. Findings suggest that there is a significant relationship between a woman's education level and the age at which she gives birth to her first child which is consistent with others [21, 30, 52–54]. In general, women who have higher levels of education tend to have their first birth at an older age compared to women with lower levels of education. This can be due to a variety of factors, such as increased access to information about reproductive health and family planning, greater economic and social opportunities, and a desire to pursue education and career goals before starting a family [5, 54–57]. Moreover, women with lower levels of education are less knowledgeable about the riskiest times to get pregnant, are less aware of family planning methods, and do not fully understand the effects of early childbearing on the health of mothers and children [58]. This finding shows that policies should prioritize the education of girls. Women can be empowered early in life with education to delay their first childbirths as a strategy for fertility reduction and maternal health improvement.

Findings suggest that age at first birth was positively correlated with age at marriage. Female adolescents who married young had their first kid sooner than those who married later. This finding concurs with prior findings [1, 21, 52]. Women who married early were more likely to give birth to their first child at an early age [2]. Moreover, women who started sexual intercourse at an early age had a higher hazard ratio of having first birth at an early age than those who started intercourse at a later age. Our study finding is in line with studies [30, 59]. Religious status shows a significant relationship with time to first birth which is consistent with prior study findings [21]. Similar results have been reported for Bangladesh; women of the Islamic faith tended to have their first child earlier than women of other beliefs [2, 60]. There are also cultural and societal factors that can influence the age at which a woman has her first birth. For example, in some communities, there may be social pressure to have children at a younger age, while in others there may be a cultural preference for having children later in life [1, 61]. Women who had no media exposure are more likely to give early birth than their counterparts. Women with access to the media can learn about family planning, reproductive health, and the detrimental consequences of early childbirth on the health of mothers and children.

Women who live in rural settings are more likely than those who live in urban areas to give birth early. Early motherhood may be a welcome alternative in rural areas when chances like school enrolment and employment opportunities are particularly scarce [21]. Moreover, the intergenerational education upward movement is higher in urban areas compared to rural areas [62]. First births are sometimes delayed depending on a woman’s residence, indicating that there are regional differences in when first births occur. This finding was consistent with earlier research conducted in Nigeria and Bangladesh [7, 21, 53]. This disparity may have been impacted by factors such as education, health awareness, and work opportunities. Moreover, findings from this study showed that women from the poorest wealth index families had a higher risk of having their first child at a young age than women from the richest families. This may be due to the fact that wealthy families are more likely to be able to pay the cost of higher education for their girls than poorer households [63]. In Bangladesh, the government has implemented various programmes and initiatives to improve access to family planning services and to promote maternal and child health [64, 65]. These efforts have helped to increase the use of modern contraception and to reduce the fertility rate in the country. However, there is still a need to continue these efforts in order to ensure that all women in Bangladesh have the information and resources they need to make informed decisions about their reproductive health.

### Strength and limitation of the study

The use of nationally representative data is the primary strength of this study. However, because the data were collected through self-report, the data's accuracy could be influenced by recall bias. The study cannot investigate the causal relationship because the used data are cross sectional. The target variable could be linked to other factors that are not investigated in this study.

## Conclusion

About 55% of the respondents’ age at their first birth was less than 18 years. Findings of survival analysis revealed that the place of residence, region, age at first marriage/sex, respondent’s education, employment status, contraceptive use, and mass media exposure were statistically significant determinants of waiting time to first birth. It is necessary to raise awareness among mothers through health education to reduce the prevalence of child marriage. Moreover, community clinics and health workers should provide proper information and counselling to mothers and families across the country about the negative impacts of early motherhood in order to enhance the health and well-being of mothers and children. The study findings will assist policymakers in identifying gaps and devising interventions aimed at reducing child mortality as well as poor maternal outcomes, which will be advantageous in Bangladesh's efforts to meet the SDGs.

## Data Availability

The data set used in this study will be available from the website of the DHS Program. After registration, one can gain access to the data files. The data set is available from the following link, http://dhsprogram.com/data/available-datasets.cfm.
